# Characterization of 42 Microsatellite Markers from Poison Ivy, *Toxicodendron radicans* (Anacardiaceae)

**DOI:** 10.3390/ijms141020414

**Published:** 2013-10-14

**Authors:** Tsai-Wen Hsu, Huei-Chuan Shih, Chia-Chi Kuo, Tzen-Yuh Chiang, Yu-Chung Chiang

**Affiliations:** 1 Endemic Species Research Institute, Nantou 552, Taiwan; E-Mail: twhsu@tesri.gov.tw; 2 Department of Nursing, Meiho University, Pingtung 912, Taiwan; E-Mails: x00002213@meiho.edu.tw (H.-C.S.); x00002077@meiho.edu.tw (C.-C.K.); 3 Department of Life Sciences, National Cheng Kung University, Tainan 701, Taiwan; 4 Department of Biological Sciences, National Sun Yat-sen University, Kaohsiung 804, Taiwan

**Keywords:** genetic diversity, microsatellite markers, poison ivy, poison oak, population structuring, *Toxicodendron*

## Abstract

Poison ivy, *Toxicodendron radicans*, and poison oaks, *T. diversilobum* and *T. pubescens*, are perennial woody species of the Anacardiaceae and are poisonous, containing strong allergens named urushiols that cause allergic contact dermatitis. Poison ivy is a species distributed from North America to East Asia, while *T. diversilobum* and *T. pubescens* are distributed in western and eastern North America, respectively. Phylogreography and population structure of these species remain unclear. Here, we developed microsatellite markers, via constructing a magnetic enriched microsatellite library, from poison ivy. We designed 51 primer pairs, 42 of which successfully yielded products that were subsequently tested for polymorphism in poison oak, and three subspecies of poison ivy. Among the 42 loci, 38 are polymorphic, while 4 are monomorphic. The number of alleles and the expected heterozygosity ranged from 1 to 12 and from 0.10 to 0.87, respectively, in poison ivy, while varied from 2 to 8 and, from 0.26 to 0.83, respectively in poison oak. Genetic analysis revealed distinct differentiation between poison ivy and poison oak, whereas slight genetic differentiation was detected among three subspecies of poison ivy. These highly polymorphic microsatellite fingerprints enable biologists to explore the population genetics, phylogeography, and speciation in *Toxicodendron*.

## 1. Introduction

*Toxicodendron radicans* (L.) Kuntze (ANACARDIACEAE), poison ivy, is a species widespread from North America to East Asia [1]. Poison ivy is a perennial woody vine with compound leaves. Urushiol, mixed oily chemical substances of pentadecylcatechols synthetized by *T. radicans* [2,3], is an allergen to humans and animals, often causing allergic contact dermatitis. Taxonomically, *T. radicans* is divided into several subspecies. For example, there are seven subspecies in North America, mostly in southern Cascades, Great Basin, and Mojave Desert [4]; in East Asia, two subspecies are distributed in Japan (ssp. *orientale*), and in Taiwan and South China (ssp. *hispidum*) [5,6]. Poison ivy is therefore a species complex consisting of many morphologically variable taxa, providing perfect materials for phylogeographic study [7,8].

In Section *Eutoxicodendron* [9], as sisters to the poison ivy, poison oaks contain two species, *T. diversilobum* and *T. pubescens* [1]. The former species are distributed in the western North America, and the latter is distributed in eastern North America. Additionally, *Toxicodendron rydbergii*, the western poison ivy, is morphological similarity but geographically distinct in western North America (1). In this study, we developed microsatellite fingerprints from the poison ivy for estimating population structuring within species (three subspecies of poison ivy) and genetic affinity among species. Theses markers are tested for the species transferability, and genetic polymorphisms.

## 2. Results and Discussion

### 2.1. Enrichment Microsatellite Library and Sequencing Results

For constructing a magnetic bead enriched library, a total of 507 white colonies were selected for sequencing from the *Toxicodendron radicans*. In total, 172 sequences were detected with microsatellite motifs that contained more than 10 repeats and 20 bps in DNA length with Tandem Repeats Finder version 4.07b [10]. Average sequence length was 818 bps, with the maximum and minimum lengths of 1496 bps and 308 bps, respectively.

### 2.2. Development of Microsatellite Markers

In total, 51 primer pairs were designed at the up- and down-flanking regions based on the primer design parameters computed with FastPCR software version 6.4.18 [11]. To test the optimal annealing temperatures, which were obtained with gradient temperature PCRs, two individuals of *Toxicodendron* species/subspecies were selected as the template DNAs. We thereby selected 42 loci from the 51 microsatellites based on unambiguous amplicoms with a gradient PCR protocol. The characteristics of 42 microsatellite loci are listed in Table 1. Of the 42 loci, 34 are complete microsatellite loci, including 23 carrying a dinucleotide motif, 5 with a trinucleotide motif, 4 with a tetranucleotide motif, and 2 with a hexanucleotide motif. Of the 8 remaining loci, 2 carried a compound motif and 6 carried interrupted motif.

### 2.3. Genotyping and Population Genetics Analysis

To examine the extent of genetic polymorphisms at each locus, 20–40 individuals were collected in fields from each subspecies of *T. radicans* (Table 2). A total of 80 plants from 3 subspecies were genotyped at the 42 microsatellite loci. Of the 42 loci, 38 loci are polymorphic and 4 are monomorphic (M67, M68, M137, and M148) in all subspecies (Table 3). In addition, two of 38 loci, AG153 and M85, cannot be amplified in ssp. *orientale* or ssp. *radicans*. To evaluate the genetic diversity, several genetic variation indices, including the number of alleles (*N*a), the effective number of alleles (*N*e), the observed and expected heterozygosities (*H*o and *H*e), and Shannon’s information index (*H*) were calculated at the 38 polymorphic loci. Here *Ne* represents an estimate of the number of equally frequent alleles in an ideal population following the formula of *N*e = 1/(1 − *H*e). As shown in Table 3, the number of alleles (*N*a) ranged from 1 to 10 in Taiwan and China populations of ssp. *hispidum*, and from 1 to 8 and 1 to 12 in ssp. *orientale* and ssp. *radicans*, respectively. *N*e varied from 1.00 to 4.82 and 1.00 to 6.78 in two areas of ssp. *hispidum*, and from 1.00 to 4.94 and 1.00 to 7.55 in two other subspecies. *H*o and *H*_E_ were also estimated in each subspecies. For example, *Ho* ranged from 0.20 to 1.00 and *H*_E_ varied 0.32 to 0.79 in Taiwan population of ssp. *hispidum*. The mean of Shannon’s information index was 0.78 in ssp. *orientale* and 0.96 in ssp. *radicans*, while it was 0.98 in the Taiwanese population and 1.07 in the mainland Chinese population of ssp. *hispidum*. Significant deviations from Hardy-Weinberg equilibrium (*H*_WE_) were detected at 1–3 loci in the subspecies of poison ivy (Table 3). A total of 27 and 7 private alleles were observed in the Taiwan and China populations of ssp. *hispidum*, respectively. Likewise, 5 private alleles were observed in ssp. *orientale* and ssp. *radicans*.

To test the transferability of these microsatellite loci, PCR amplification was conducted on these primers in two species of the poison oaks, including *T. diversilobum* and *T. pubescens*. In total, 20 samples from three populations of each species were used for the cross-species amplification (Table 4). Of 42 loci, 25 loci were of successful transferability. At these polymorphic loci, *Na* and *Ne* ranged from 2 to 8 and from 1.60 to 5.80 in *T. diversilobum*, and from 2 to 8 and from 1.34 to 5.56 in *T. pubescens* (Table 4). *H*o and *H*_E_ ranged from 0.35 to 0.85 and 0.38 to 0.83 in *T. diversilobum* and 0.30 to 0.90 and 0.26 to 0.82 in *T. pubescens*, respectively. The average of Shannon’s information index of 0.69 and 0.61 was observed in *T. diversilobum* (with 12 private alleles) and *T. diversilobum* (with one single private allele), respectively. No loci were detected with significant deviations from *H*_WE_ in the poison oak, except for two loci in *T. pubescens*.

**Figure 1:**
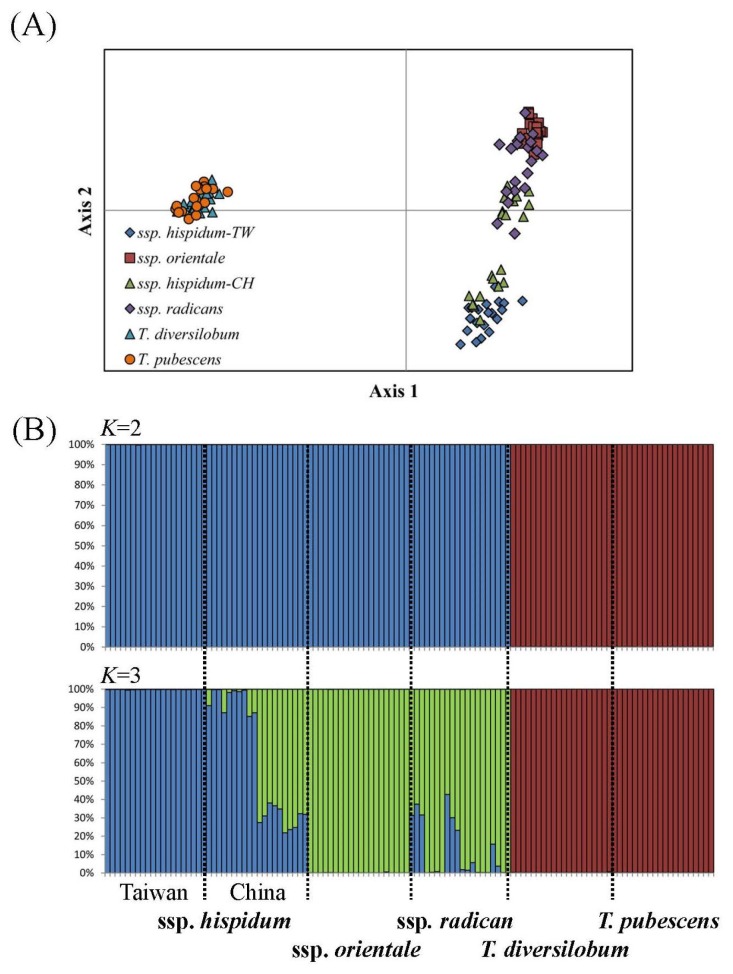
(**A**) Plots of the first two axes in principle coordinate analysis (PCoA) and (**B**) the assignment test with Bayesian clustering analysis, including the best (*K* = 2) and second fit numbers (*K* = 3) of grouping based on 38 polymorphic microsatellite loci. Abbreviations TW and CH indicate Taiwan Island and mainland China, respectively.

Genetic composition and distinction within and between *Toxicodendron* taxa was examined with a principle coordinate analysis (PCoA) and Bayesian assignment test (Figure 1). Based on 38 polymorphic microsatellite loci, the genetic composition of poison ivy was differentiated from that of the poison oak, as indicated by the first axis, which explained 58.41% variation (Figure 1A). Within poison oaks, the genetic composition cannot be distinguished at the first or second axis (Figure 1A), indicating genetic homogeneity without geographic differentiation. Among subspecies within *T. radicans*, genetic compositions among subspecies cannot be separated at the first axis but are spread out by the second axis (explained 21.69% variations) (Figure 1A). Subspecies of the poison ivy were not significantly differentiated as indicated by PCoA, a pattern similar to the results based on ISSR fingerprints [6].

Clustering of poison ivy and poison oak was examined with STRUCTURE analysis [12–14]. The best and second fit numbers of grouping were inferred as two and three by the Δ*K* evaluations (Δ*K* = 216.171 at *K* = 2 and Δ*K* = 157.323 at *K* = 3) based on the Bayesian assignment test. When *K* = 2, *Toxicodendron* taxa were divided into two major groups (Figure 1B). The first and second groups with a high percentage of composition 1 (segment in blue, Figure 1B) or composition 2 (segment in red, Figure 1B) corresponded to the poison oak and ivy, respectively. When *K* = 3, composition 1 (*T. radicans*) was subdivided into composition 1a (blue segment in Figure 1B) and 1b (green segment in Figure 1B). Several individuals from China of ssp. *hispidum* and of ssp. *radicans* displayed genetic admixture, likely due to shared ancestral polymorphism [15] or recurrent gene flow [16,17].

## 3. Experimental Section

### 3.1. Sampling and DNA Extractions

Twenty individuals were collected from three populations of the poison oak (*T. pubescens*, *T. diversilobum*), and of each subspecies of poison ivy, *T. radicans subsp. orientale*, and ssp. *hispidum* from Taiwan and mainland China, respectively (Table 2). The sample size, location, and voucher specimens number are listed in Table 2. All voucher specimens were deposited in the Herbarium of Taiwan Endemic Species Research Institute (TAIE). Total genomic DNAs were extracted from silica-dried leaf powder using the Plant Genomic DNA Extraction Kit (RBC Bioscience, Taipei, Taiwan).

### 3.2. Isolation of Microsatellite DNA Loci and Identification

The modified AFLP [18] and magnetic bead enrichment method [19,20] were used to select microsatellite loci. Genomic DNA of *T. radicans* ssp. *radicans* was digested by restriction enzyme *Mse*I (Promega, Madison, WI, USA) and electrophoresed on 1% Nusieve^®^ 3:1 agarose gels (FMC Bio Products, Rockland, ME, USA). Fragment DNAs of 400 to 1000 bp were isolated using HiYield™ Gel PCR DNA Fragments Extraction Kit (RBC Bioscience) and ligated to a double stranded *Mse*I-adaptor (complementary oligo A: 5′-TACTCAGGACTCAT-3′, 5′ phosphorylated oligo B: 5′-GACGATGAGTCCTGAG-3′) and incubated at 21 °C overnight. Ligated products were used as template DNAs for prehybridization PCR in order to enrich the partial genomic library. Total 20 μL of PCR cocktail was included with 20 ng template DNA, 10 pmol adapter-specific primer (5′-GATGAGTCCTGAGTAAN-3′), 2 μL 10× reaction buffer, 2 mM dNTP mix, 2 mM MgCl_2_, 0.5 U Taq DNA polymerase (Promega), and sterile water. The amplification reaction was executed at 94 °C for 5 min, followed by 18 cycles of 94 °C for 30 s, 53 °C for 1 min, and 72 °C for 1 min using a Labnet MultiGene 96-well Gradient Thermal Cycler (Labnet, Edison, NJ, USA). PCR products were denatured and hybridized to two biotinylated probes (B-(AG)_15_, B-(AC)_15_) at 68 °C for 1 h, followed by addition of 1 mg Streptavidin MagneSphere Paramagnetic Particles (Promega) to capture the hybridizations at 42 °C for 2 h. The enriched DNA fragments were eluted with high- and low-salt solutions and used as template DNAs for 25 cycles of PCR amplification. PCR cocktail and amplifciation protocol were identical to prehybridization PCR except the number of PCR cycles. Amplicons were purified using HiYield™ Gel PCR DNA Fragments Extraction Kit (RBC Bioscience) and then cloned using pGEM^®^-T Easy Vector System (Promega). White colonies were selected and screened using PCR with primer pairs: (AG)_10_ or (AC)_10_/SP6 or T7). Selected clones were purified and sequenced in both directions with an ABI PRISM^®^ 3700 DNA Sequencer (Applied Biosystems, Inc., Foster City, CA, USA.). Sequences containing tandem repeat sequences were identified using Tandem Repeats Finder version 4.07b [10]. We designed the pair of specific primers for each microsatellite locus using FastPCR software version 6.4.18 [11]. The parameters for the microsatellite specific primer design were set at a PCR product size ranging from 100 to 450 bp, an optimum annealing temperature of 55 °C, and a GC content ranging from 40% to 60%.

### 3.3. DNA Amplification and Genotyping

For testing annealing temperature, each primer pair was evaluated following a gradient PCR procedure. All primer pairs were tested for PCR amplification on DNA extracted from each species and subspecies, *i.e*., one individual of *T. pubescens*, *T. diversilobum*, *T. radicans* subsp. *radicans*, and two individuals of *T. radicans* subsp. *orientale*, and *T. radicans* subsp. *hispidum* from Taiwan and mainland China. The procedure was performed at 94 °C for 5 min, followed by 30 cycles of 94 °C for 40 s, 48–65 °C for 60 s, 72 °C for 60 s, and a final extension of 72 °C for 10 min with the Labnet MultiGene 96-well Gradient Thermal Cycler (Labnet). Amplicoms were checked by 10% PAGE electrophoresis to separate the target DNA bands, which were confirmed based on sequences. These SSR primer pairs with confirmed target DNA bands were chosen for polymorphism evaluation.

For examining genetic polymorphisms, 20 individuals from 3 populations of two species and four subspecies (Table 2) were selected. PCR amplifications were performed using a Labnet MultiGene 96-well Gradient Thermal Cycler (Labnet), in a 20 μL reaction cocktail containing 20 ng template DNA, 0.2 μM each of reverse and forward primers, 2 μL 10× reaction buffer, 2 mM dNTP mix, 2 mM MgCl_2_, 0.5 U Taq DNA polymerase (Promega), and sterile water. The PCR program was conducetd at 94 °C for 5 min, followed by 30 cycles of 94 °C for 40 s, at the optimal annealing temperature (*T*a) for 60 s, 72 °C for 60 s, and a final extension of 72 °C for 10 min [21]. Amplicons were visualized under UV light by electrophoresis on a 10% polyacrylamide gel (acrylamide: bisacrylamide 29:1, 80 V for 14–16 h) using a 25 or 50 bp DNA Step Ladder (Promega) to determine the allele size. The sizes of the PCR products were detected and analyzed using Quantity One software version 4.62 (Bio-Rad Laboratories, Hercules, CA, USA).

### 3.4. Data Analysis

Genetic variation indices, including the number of alleles (*N*a), the effective number of alleles (*N*e), the observed and expected heterozygosity (*H*o and *H*e), Shannon’s information index, private alleles were calculated using GenAlEx version 6.4 [22]. Hardy–Weinberg equilibrium (*H*_WE_) was tested using Arlequin software version 3.5.1.2 [23].

Genetic composition and genetic distinction among *Toxicodendron* species and subspecies were evaluated using the PCoA by GenAlEx version 6.4 [22] and the Bayesian assignment test using STRUCTURE version 2.3.3 [12–14]. The posterior probability of the grouping number (*K* = 1~6) was calculated by the Markov chain Monte Carlo (MCMC) method with 20 separate runs to estimate the stability of the results. Each run was assessed with 5,000,000 steps and a 500,000-step burn-in based on the admixture model [24]. The best fit number of grouping was assessed by Δ*K* [25] using STRUCTURE HARVESTER version 0.6.8 [26]. A final 10,000,000 simulation with a 1,000,000-step burn-in was performed based on the best *K*.

## 4. Conclusions

In total 42 microsatellite loci, including 38 polymorphic and 4 monomorphic, developed from *Toxicodendron radicans* are characterized in two species of the poison oak and three subspecies of the poison ivy. These SSR fingerprints were useful in assessing the population structuring and genetic diversity in taxa from different geographic areas. Genetic analyses revealed significant differentiation between poison oaks and poison ivy, whereas slight differentiation was seen among subspecies of the poison ivy. Furthermore, abundant allelic polymorphisms in these microsatellite fingerprints make them useful for genetic assessing genetic diversity, population differentiation, phylogeography, and speciation.

## Figures and Tables

**Table 1:** Characteristics of 42 microsatellite loci isolated from *Toxicodenderon radicans*.

Locus	Primer sequence (5′–3′ )	Repeat motif	Allelic size (bp)	*Ta* (°C)	GenBank Accession No.
AC3	F: GCGCAAATACGAAAGCGAGA	(AG)_27_	104~146	55	HF680270
	R: AAAAATGGGCTCAAGCGATC				

AC6	F: CGGGATCGATGATGAGTCCTGA	(ATT)_7_(TTC)_2_N(CTT)_13_	299~337	55	HF680271
	R: ATCAGAGGAGCGAGTCAGC				

AC11	F: GTGAAGAAACTGAAGAGCCAC	(AG)_24_	194~218	55	HF680272
	R: TCACCAAAACTTAAGGGTGG				

AC19	F: CCACTCCACCCGTAACAACG	(AGAAAA)_5_N(CT)_14_N(ATG)_7_	324~340	55	HF680273
	R: TCGTCCGTCATCGCTGCCCT				

AC20	F: CGTGCGTTACTTCTGCTCAC	(ATG)_12_(AAG)_9_(ATG)_9_	237~245	55	HF680274
	R: ACTGTGAATCACCTGACCACG				

AC139	F: GAGGTGATATTGGTACTTGG	(TA)_9_(GA)_10_	112~128	55	HF680275
	R: TTCCTCTCACTTTTACGTTC				

AG28	F: TATCGCATCAGGGGTTCCCA	(GGA)_15_	222~230	55	HF680276
	R: CGGGATGGAGCCGCCAATGA				

AG153	F: GATGAGTCCTGAGTAAACCA	(TTTC)_19_	165–169	51	HF680277
	R: TGCATATTTCATGATAATGG				

M8	F: TTCTTCTTCATTGTGCCGTC	(GA)_23_	136~140	55	HF680278
	R: ATGTAGGCATGAATGAGGTG				

M18	F: AGGCTCCAAATCCATGCCTC	(AAGA)_27_	187~195	55	HF680279
	R: CAAGAGCAAGAACATAGAATATAA				

M19	F: AGTGAATAGGTAGAATTCTCC	(AG)_22_	129~129	55	HF680280
	R: CGGATTTTAGCTCAATTCCATC				

M22	F: AAGGATCAAGAAGGAAGGTG	(AG)_30_	155~159	55	HF680281
	R: CCCTTCTCTTTCTTCTTCCC				

M24	F: GATTCATCTGGGTCACCTGG	(GAGTGA)_14_	166~178	55	HF680282
	R: GACAATAGACTCCGACAACG				

M27	F: CATTCTTCTTCATTGTGCCG	(GA)_27_	110~112	55	HF680283
	R: CCAATTTACCGAATCCAAGC				

M30	F: AAAGTTCATCATGGGTGTTTG	(TG)_16_	124~148	55	HF680284
	R: AAACAAATCAGCCCTTCCAC				

M31	F: AGTTGTGTATGTCTGTGTTG	(GT)_92_	218~244	55	HF680285
	R: AAACAAAGATGATGTAAAACGC				

M452	F: GACCAAGTGAAGCTGAATAG	(GA)_12_	75~105	55	HF680286
	R: CTCACCAACTCAGCTAAGC				

M493	F: GCATCCTTCATTTTCTTATGG	(AAGA)_25_	221~223	55	HF680287
	R: CGTTATCCAAACAACTCCAC				

M54	F: AAAACGTTAGCCGATAAGG	(GA)_15_	108~132	55	HF680288
	R: TCAGCCTCTCCCCTCTTTTC				

M56	F: TGGAGATGGAGATGAAGAGG	(AG)_12_	93~123	55	HF680289
	R: GCGTAAGATAGTCACTGTAC				

M60	F: AACTGAAGAGGTGCAATGGG	(TGA)_17_	122~144	55	HF680290
	R: AGAGACTCTTCATCTTCTCC				

M61	F: CCGTTCACTGATTTTGCTAG	(AG)_11_	169~207	55	HF680291
	R: CTGGCTACTAGATGATCCAG				

M64	F: ATAGTGAGTGCATGGTGGCG	(AG)_17_	114~128	55	HF680292
	R: CTCCTCTTGAAACTGAGCTG				

M66	F: TGGAGCACTCATTTGTAACG	(AG)_11_N(AG)_9_N(AG)_9_	116~132	55	HF680293
	R: CTGGATCTATACTCAATTCC				

M67	F: AGTGTGCTCTAAGAGTAAGG	(GAAT)_14_	153	55	HF680294
	R: TATCCTACTAGGACTCTACC				

M68	F: CTGGTGTTGGGAAAGAAGG	(TGGTGA)_10_	120	51	HF680295
	R: TTATTACCATATTATCCTTTACAT				

M821	F: TTGTCATCGTCGTCCAAACC	(TTG/A)_11_	158~160	55	HF680296
	R: AAATCTCCTCATCCAACGCC				

M822	F: GGTGGATTGAAGAAATGACG	(GA)_4_(GAGAA)_4_N(GA)_12_	127~149	55	HF680297
	R: AAATTCATTCGCTTTCACCTC				

M83	F: CATTCAACGCCGACAATTCC	(AAT/C)_16_	124~126	55	HF680298
	R: TCCATATTCAGCCCAAGTGC				

M85	F: TTTGCTTTGGTTGAGAGTGC	(AG)_11_	118~122	55	HF680299
	R: AATGTAATGTTCCTCCAACG				

M97	F: AGTTCTGGAGCTCAACATGG	(GT)_12_	163~179	55	HF680300
	R: TCGAAGCTCTGATACCACTG				

M99	F: CCTTCCGGAGAGGTAGATTG	(AG)_10_	140~152	55	HF680301
	R: TCTATAAGTACACCTTCTCC				

M104	F: TGGATTAGGCGAGTCACACC	(AG)_15_	149~157	55	HF680302
	R: GTTTCACAGCATCCACGTGC				

M120	F: CGACTCATAATTGACGAGCC	(TG)_10_	119~143	55	HF680303
	R: CTGTAAAATTACTATAGCCC				

M121	F: TGATTCTTTTGTGGTTTGCG	(AG)_14_	210~216	55	HF680304
	R: TGTGTAGTGATTATAGAAGG				

M123	F: GTAATGTGTTTCAGTGCGTC	(AG)_12_	138~154	55	HF680305
	R: CTTTTGGGCTATCATGGATG				

M124	F: AAGTACAGTTCCCGAAACTG	(AAAG)_10_N(AG)_11_	296~320	55	HF680306
	R: TATTTTCACTAACCCTACCC				

M137	F: AGTGAGCTATCCAGCTATCG	(AG)_22_	124	52	HF680307
	R: TCGTGTCAGTTTCGAGTAGC				

M148	F: GATCTGAATTTTCCGAAAGCG	(AG)_10_	197	53	HF680308
	R: AGTGGGAGTTACAGTATACC				

M154	F: AAGAACTTCATTCACCGTCC	(TGG)_102_	417~445	55	HF680309
	R: GTACTGCCTTCAAGGAAGTC				

M155	F: TCTAACCCTTCCAAAATTGG	(AG)_12_	130~147	55	HF680310
	R: AAATTATGGGCCTGTTACTG				

M156	F: AAGCTAGCAAATACACATAGG	(CA)_14_(CT)_9_N(AAT/C)_16_	120~152	55	HF680311
	R: CTGACAAGTTCCAGACAGGG				

Note: F = the forward primer; R = the reverse primer; *T*a = optimized annealing temperature.

**Table 2:** Sample location for each species, subspecies, and populations of *Toxicodenderon*. Sample size, location, coordinate, and voucher specimens are indicated.

Species	Locality	Sample size	Longitude	Latitude	Voucher Specimens Number
*T. radicans* ssp*. hispidum*	Yilan Co., Taiwan	7	N 24°30′26.2″	E121°31′00.7″	Hsu18286
	Hsinchu, Taiwan	7	N 24°30′17.0″	E121°07′05.6″	Hsu18285
	Nantou, Taiwan	6	N 24°06′39.8″	E121°12′50.5″	Hsu18287

ssp. *hispidum*	Daguan, Yunnan, China	7	N 28°12′28.6″	E103°56′26.8″	Hsu18290
	Leibo, Sichuan, China	6	N 28°20′50.4″	E103°43′49.8″	Hsu18289
	Pingshan, Sichuan, China	7	N 28°43′31.1″	E103°58′09.7″	Hsu18295

ssp*. orientale*	Kochi, Shikoku, Japan	7	N 33°46′02.4″	E134°02′11.0″	Hsu18281
	Okayama, Honshu, Japan	7	N 35°05′18.1″	E133°31′35.6″	Hsu18282
	Nagano, Honshu, Japan	6	N 36°10′59.6″	E137°31′30.0″	Hsu18284

ssp*. radicans*	Washington Co., MO, USA	7	N 38°04′20.1″	W90°41′57.6″	Hsu18300
	Montgomery Co., MO, USA	7	N 38°51′25.9″	W91°30′57.6″	Hsu18296
	Monroe Co., MO, USA	6	N 39°30′50.3″	W91°47′24.1″	Hsu18298

*T. diversilobum*	Butte Co., CA, USA	7	N 39°32′08.5″	W121°25′24.4″	Hsu18302
	Chico, CA, USA	7	N 39°44′06.9″	W121°49′38.1″	Hsu18303
	Medford, OR, USA	6	N 42°17′34.9″	W122°49′55.3″	Hsu18305

*T. pubescens*	Carter Co., MO, USA	7	N 36°55′40.5″	W91°07′12.5″	Hsu18304
	Oregon Co., MO, USA	7	N 36°48′29.8″	W91°07′45.3″	Hsu18306
	Howell Co., MO, USA	6	N 36°32′23.9″	W91°50′29.7″	Hsu18307

**Table 3:** Average genetic diversity for three subspecies of *Toxicodenderon radicans* based on the 42 newly developed microsatellites. For each locus, number of alleles (*N*a), effective number of alleles (*N*e), observed heterozygosity (*H*_O_), expected heterozygosity (*H*_E_), and Shannon’s information index (*H*) are indicated.

Locus	ssp. *hispidum* (Taiwan)					ssp. *hispidum* (China)					ssp. *orientale*					ssp. *radicans*				

	*N*a	*N*e	*H*_O_	*H*_E_	*H*	*N*a	*N*e	*H*_O_	*H*_E_	*H*	*N*a	*N*e	*H*_O_	*H*_E_	*H*	*N*a	*N*e	*H*_O_	*H*_E_	*H*
AC3	6	3.77	0.65	0.74	1.45	10	6.78	0.70	0.85	2.04	8	4.94	0.80	0.80	1.76	8	5.23	0.60	0.81	1.78
AC6	4	2.62	0.60	0.62	1.14	9	5.44	0.80	0.82	1.88	7	4.60	0.80	0.78	1.68	12	7.55	0.85	0.87	2.19
AC11	3	2.23	0.35	0.55	0.89	6	3.76	0.50	0.73	1.49	7	4.08	0.55	0.76	1.58	6	3.46	0.45	0.71 *	1.39
AC19	3	2.04	0.30	0.51	0.78	5	3.49	0.45	0.71	1.37	6	4.62	0.60	0.78	1.62	7	4.44	0.85	0.78	1.64
AC20	3	2.07	0.25	0.52	0.78	3	2.35	0.50	0.57	0.94	3	2.35	0.50	0.57	0.94	4	2.69	0.60	0.63	1.11
AC139	2	1.66	0.35	0.40	0.59	3	1.97	0.45	0.49	0.85	3	2.57	0.65	0.61	1.02	3	2.56	0.55	0.61	1.02
AG28	4	3.24	0.50	0.69 *	1.23	3	2.17	0.40	0.54	0.90	3	2.27	0.45	0.56 *	0.90	3	2.51	0.45	0.60	0.98
AG153	3	1.87	0.30	0.47 *	0.82	2	1.72	0.30	0.42	0.61	-	-	-	-	-	-	-	-	-	-
M8	2	1.47	0.20	0.32	0.50	2	1.22	0.10	0.18 *	0.33	1	1.00	-	-	-	1	1.00	-	-	-
M18	4	3.52	0.85	0.72	1.32	3	2.33	0.60	0.57	0.96	1	1.00	-	-	-	3	1.68	0.40	0.41	0.74
M19	2	1.72	0.30	0.42	0.61	1	1.00	-	-	0.00	1	1.00	-	-	-	1	1.00	-	-	-
M22	2	1.66	0.25	0.40	0.59	3	1.68	0.25	0.41	0.74	2	1.88	0.35	0.47	0.66	2	1.96	0.35	0.49	0.68
M24	2	1.78	0.25	0.44	0.63	3	2.85	0.50	0.65	1.07	4	3.77	0.70	0.74	1.35	5	4.65	0.60	0.79	1.57
M27	2	1.78	0.35	0.44	0.63	2	1.28	0.15	0.22	0.38	1	1.00	-	-	-	1	1.00	-	-	-
M30	4	3.85	0.90	0.74	1.37	6	4.02	0.85	0.72	1.52	4	3.83	0.80	0.74	1.36	8	4.82	0.75	0.79	1.77
M31	5	3.90	0.55	0.74 *	1.45	8	5.63	0.65	0.82	1.88	1	1.00	-	-	-	4	1.80	0.35	0.44	0.86
M452	3	2.30	0.40	0.57	0.93	3	1.11	0.10	0.10	0.23	4	1.78	0.35	0.44	0.82	5	1.68	0.60	0.41	0.80
M493	2	1.72	0.30	0.42	0.61	2	1.60	0.30	0.38	0.56	1	1.00	-	-	-	1	1.00	-	-	-
M54	6	4.79	0.75	0.79	1.67	6	3.88	0.70	0.74	1.56	6	3.56	0.70	0.72	1.45	6	3.54	0.50	0.72 *	1.47
M56	1	1.00	-	-	0.00	5	2.42	0.45	0.59	1.16	5	3.62	0.50	0.72	1.42	6	3.16	0.55	0.68	1.41
M60	6	4.65	1.00	0.79	1.65	6	3.98	0.75	0.75	1.59	2	1.83	0.30	0.46	0.65	5	2.17	0.50	0.54	1.09
M61	2	1.92	0.40	0.48	0.67	6	4.12	0.75	0.76	1.57	7	3.90	0.85	0.74	1.60	8	5.52	0.80	0.82	1.86
M64	4	3.38	0.55	0.70	1.27	3	2.60	0.60	0.62	1.01	3	2.06	0.40	0.52	0.82	3	1.80	0.45	0.45	0.75
M66	2	1.88	0.45	0.47	0.66	3	2.46	0.55	0.59	0.97	1	1.00	-	-	-	2	1.78	0.35	0.44	0.63
M67	1	1.00	-	-	-	-	-	-	-	-	-	-	-	-	-	-	-	-	-	-
M68	1	1.00	-	-	-	1	1.00	-	-	-	1	1.00	-	-	-	1	1.00	-	-	-
M821	2	1.78	0.45	0.44	0.63	2	1.47	0.30	0.32	0.50	1	1.00	-	-	-	1	1.00	-	-	-
M822	1	1.00	-	-	0.00	2	1.16	0.15	0.14	0.27	2	1.91	0.35	0.48	0.81	4	1.95	0.40	0.49	0.89
M83	2	1.98	0.50	0.50	0.69	2	1.41	0.25	0.29	0.46	2	1.28	0.15	0.22	0.38	2	1.28	0.15	0.22	0.38
M85	3	2.97	0.40	0.66	1.09	3	2.52	0.50	0.60	1.00	-	-	-	-	-	-	-	-	-	-
M97	5	3.52	0.75	0.72	1.37	5	3.76	0.60	0.73	1.45	1	1.00	-	-	-	4	2.03	0.35	0.51	0.98
M99	6	4.08	0.50	0.76	1.51	4	3.29	0.60	0.70	1.28	2	1.47	0.20	0.32	0.50	2	1.96	0.35	0.49	0.68
M104	5	3.86	0.60	0.74	1.46	3	2.63	0.40	0.62 *	1.03	1	1.00	-	-	-	2	1.72	0.30	0.42	0.61
M120	7	4.82	0.70	0.79	1.73	6	5.06	0.70	0.80	1.71	4	2.79	0.60	0.64	1.15	5	2.56	0.55	0.61	1.19
M121	3	2.47	0.50	0.60	1.00	3	2.69	0.55	0.63	1.04	2	1.72	0.30	0.42	0.61	3	2.38	0.70	0.58	0.94
M123	3	2.69	0.55	0.63	1.04	6	3.24	0.65	0.69	1.46	7	4.76	0.60	0.79	1.70	7	3.96	0.80	0.75	1.63
M124	6	3.13	0.65	0.68	1.35	4	3.01	0.65	0.67	1.17	3	2.35	0.40	0.57	0.94	4	2.78	0.60	0.64	1.17
M137	1	1.00	-	-	-	1	1.00	-	-	-	1	1.00	-	-	-	1	1.00	-	-	-
M148	1	1.00	-	-	-	1	1.00	-	-	-	1	1.00	-	-	-	1	1.00	-	-	-
M154	3	2.97	0.60	0.66	1.09	4	2.91	0.60	0.66	1.18	5	3.03	0.45	0.67	1.27	6	3.94	0.65	0.75	1.48
M155	3	2.75	0.40	0.64	1.06	4	2.68	0.45	0.63	1.15	4	2.91	0.50	0.66	1.19	4	2.67	0.50	0.63	1.17
M156	7	1.83	0.40	0.45	1.01	7	2.95	0.65	0.66 *	1.39	6	1.47	0.65	0.74	1.47	7	3.15	0.65	0.68	1.42

Mean	3.26	2.49	0.49	0.59	0.98	3.93	2.73	0.50	0.59	1.07	3.18	2.27	0.52	0.61	0.78	4.05	2.60	0.53	0.60	0.96

* Significance of deviation from Hardy-Weinberg equilibrium: *p* < 0.05.

**Table 4:** Average genetic diversity in poison oak, *Toxicodendron diversilobum* and *T. pubescens*, at 42 loci with high transferability. For each locus, number of alleles (*N*a), effective number of alleles (*N*e), observed heterozygosity (*H*_O_), expected heterozygosity (*H*_E_), and Shannon’s information index (*H*) are indicated.

Locus	*T. diversilobum*					*T. pubescens*				
	
	*N*a	*N*e	*H*_O_	*H*_E_	*H*	*N*a	*N*e	*H*_O_	*H*_E_	*H*
AC3	6	3.94	0.70	0.75	1.54	6	3.94	0.65	0.75	1.54
AC6	5	4.62	0.80	0.78	1.56	5	4.71	0.80	0.79	1.58
AC11	8	5.80	0.85	0.83	1.90	8	5.56	0.90	0.82	1.87
AC19	5	3.69	0.80	0.73	1.45	6	3.08	0.65	0.68	1.43
AC20	2	1.96	0.35	0.49	0.68	2	1.96	0.45	0.49	0.68
AC139	-	-	-	-	-	-	-	-	-	-
AG28	4	3.29	0.50	0.70	1.28	3	2.82	0.40	0.65	1.07
AG153	-	-	-	-	-	-	-	-	-	-
M8	-	-	-	-	-	-	-	-	-	-
M18	5	2.83	0.60	0.65	1.25	4	3.92	0.50	0.75 *	1.38
M19	3	1.94	0.35	0.48	0.83	1	1.00	-	-	-
M22	3	1.94	0.35	0.48	0.83	3	2.52	0.45	0.60	1.00
M24	-	-	-	-	-	-	-	-	-	-
M27	2	2.00	0.40	0.50	0.69	2	1.98	0.40	0.50	0.69
M30	6	2.74	0.60	0.64	1.26	3	2.20	0.55	0.55	0.86
M31	-	-	-	-	-	-	-	-	-	-
M452	1	1.00	-	-	-	1	1.00	-	-	-
M493	-	-	-	-	-	-	-	-	-	-
M54	-	-	-	-	-	-	-	-	-	-
M56	-	-	-	-	-	-	-	-	-	-
M60	7	5.30	0.80	0.81	1.77	4	2.71	0.65	0.63 *	1.15
M61	5	3.40	0.60	0.71	1.39	2	1.34	0.30	0.26	0.42
M64	-	-	-	-	-	-	-	-	-	-
M66	2	1.60	0.30	0.38	0.56	2	1.98	0.30	0.50	0.69
M67	-	-	-	-	-	-	-	-	-	-
M68	-	-	-	-	-	-	-	-	-	-
M821	2	1.98	0.40	0.50	0.69	2	1.98	0.40	0.50	0.69
M822	2	1.60	0.50	0.38	0.56	2	1.60	0.40	0.38	0.56
M83	2	1.96	0.45	0.49	0.68	2	1.98	0.50	0.50	0.69
M85	1	1.00	-	-	-	1	1.00	-	-	-
M97	-	-	-	-	-	-	-	-	-	-
M99	-	-	-	-	-	-	-	-	-	-
M104	6	4.85	0.80	0.79	1.67	5	4.19	0.60	0.76	1.49
M120	3	2.06	0.45	0.52	0.89	2	1.83	0.30	0.46	0.65
M121	-	-	-	-	-	-	-	-	-	-
M123	5	2.29	0.55	0.56	1.13	5	2.32	0.55	0.57	1.15
M124	5	4.28	0.65	0.77	1.53	5	4.19	0.70	0.76	1.52
M137	-	-	-	-	-	-	-	-	-	-
M148	-	-	-	-	-	-	-	-	-	-
M154	3	2.38	0.45	0.58	0.94	3	2.22	0.40	0.55	0.92
M155	3	2.69	0.60	0.63	1.04	3	2.52	0.60	0.60	1.00
M156	-	-	-	-	-	-	-	-	-	-

Mean	3.84	2.85	0.56	0.62	0.69	3.28	2.58	0.52	0.58	0.61

* Deviation from Hardy-Weinberg equilibrium: *p* < 0.05.
